# Cortical Sensorimotor Processing of Painful Pressure in Patients with Chronic Lower Back Pain—An Optical Neuroimaging Study using fNIRS

**DOI:** 10.3389/fnhum.2016.00578

**Published:** 2016-11-17

**Authors:** Andrea Vrana, Michael L. Meier, Sabina Hotz-Boendermaker, Barry K. Humphreys, Felix Scholkmann

**Affiliations:** ^1^Interdisciplinary Spinal Research, Department of Chiropractic Medicine, University Hospital BalgristZürich, Switzerland; ^2^Department of Health Sciences and Technology, Human Movement Sciences and Sport, ETH ZürichZürich, Switzerland; ^3^Biomedical Optics Research Laboratory (BORL), Department of Neonatology, University Hospital ZürichZürich, Switzerland

**Keywords:** functional near-infrared spectroscopy, back pain, hemodynamic response, sensorimotor cortex, chronic pain

## Abstract

In this study we investigated sensorimotor processing of painful pressure stimulation on the lower back of patients with chronic lower back pain (CLBP) by using functional near-infrared spectroscopy (fNIRS) to measure changes in cerebral hemodynamics and oxygenation. The main objectives were whether patients with CLBP show different relative changes in oxy- and deoxyhemoglobin ([O_2_Hb] and [HHb]) in the supplementary motor area (SMA) and primary somatosensory cortex (S1) compared to healthy controls (HC). Twelve patients with CLBP (32 ± 6.1 years; range: 24–44 years; nine women) and 20 HCs (33.5 ± 10.7 years; range 22–61 years; eight women) participated in the study. Painful and non-painful pressure stimulation was exerted with a thumb grip perpendicularly to the spinous process of the lumbar spine. A force sensor was attached at the spinous process in order to control pressure forces. Tactile stimulation was realized by a one-finger brushing. Hemodynamic changes in the SMA and S1 were measured bilaterally using a multi-channel continuous wave fNIRS imaging system and a multi-distant probe array. Patients with CLBP showed significant stimulus-evoked hemodynamic responses in [O_2_Hb] only in the right S1, while the HC exhibited significant [O_2_Hb] changes bilaterally in both, SMA and S1. However, the group comparisons revealed no significant different hemodynamic responses in [O_2_Hb] and [HHb] in the SMA and S1 after both pressure stimulations. This non-significant result might be driven by the high inter-subject variability of hemodynamic responses that has been observed within the patients group. In conclusion, we could not find different stimulus-evoked hemodynamic responses in patients with CLBP compared to HCs. This indicates that neither S1 nor the SMA show a specificity for CLBP during pressure stimulation on the lower back. However, the results point to a potential subgrouping regarding task-related cortical activity within the CLBP group; a finding worth further research.

## Introduction

Lower back pain (LBP) affects between 70–85% of people worldwide at least once in their lifetime (Andersson, 1997; Balagué et al., 2012). Within the Swiss population, 47% of women and 39% of men are suffering from back pain longer than 4 weeks (Lloyd et al., 2008). The prognosis for not developing chronic lower back pain (CLBP) is good with about 90% of patients recovering within a few days or weeks. However, 5–10% of patients become chronic (pain lasts >3 months), with often disabling pain causing additional severe interferences with everyday living and working activities (Blumer and Heilbronn, 1982; Brown, 1990; Andersson, 1999). Despite this high incidence and prevalence of LBP, the factors responsible are not well understood (Andersson, 1999). In the majority of chronic cases, a clearly attributable pathology is lacking (Balagué et al., 2012). Therefore, CLBP has mostly a non-specific origin, which considerably impedes the search for causes and treatments.

Within the last two decades, the search for causes and/or consequences of CLBP has been extended to the brain. Structural, neurochemical and functional maladaptive alterations have been reported in several cortical areas, i.e., the insula, anterior cingulate cortex (ACC), thalamus, primary and secondary somatosensory cortices (S1, S2) and also prefrontal cortices and the cerebellum (Flor et al., 1997; Peyron et al., 2000; Apkarian et al., 2005; Schmidt-Wilcke et al., 2006; Tracey and Mantyh, 2007; Tsao et al., 2008; Wand et al., 2011). However, the findings regarding maladaptive alterations in patients with CLBP are ambiguous. Some reports show decreased cortical activity in patients with CLBP during pain processing in regions such as the thalamus, S1, S2 and ACC (Peyron et al., 2000; Apkarian et al., 2005; Jensen et al., 2016). Others have found augmented cortical activity compared to healthy controls (HC) in S1, S2, cerebellum and parietal cortices (Giesecke et al., 2004) or the right insula, supplementary motor area (SMA) and posterior cingulate cortex (PCC; Kobayashi et al., 2009) after painful stimulation. This ambiguity of results might be also explained by the sparse evidence about the cortical pain processing mechanisms in healthy; recent investigations do not corroborate pain-specificity of the so-called “pain matrix” but rather suggest a saliency-related activation (Iannetti and Mouraux, 2010; Legrain et al., 2011; Üçeyler et al., 2015).

It has been previously reported that patients with CLBP can exhibit sensorimotor maladaptive alterations at the peripheral level like decreased two-point-discrimination, decreased postural coordination and an impaired or disrupted body schema (Hodges, 2001; Coslett et al., 2002; Moseley, 2008; Jacobs et al., 2009; Wand et al., 2010, 2013; Luomajoki and Moseley, 2011). Despite these manifold peripheral sensorimotor changes, only a few former investigations have addressed the cortical sensorimotor processing in CLBP patients in particular (Flor et al., 1997; Lloyd et al., 2008; Tsao et al., 2008, 2011; Kobayashi et al., 2009), revealing mainly shifts in the representation of body parts. However, the sensorimotor system might be affected not only with respect to the representation but also regarding the intensity of recruitment or activity as it shows a high level of neural plasticity (Boudreau et al., 2010a); it might therefore be prone to (maladaptive) alterations due to the recurrent pain in CLBP. Therefore, in the current investigation, the focus was laid on solely investigating the sensorimotor processing and its potential reorganization in patients with CLBP. In order to investigate the sensorimotor processing in patients with CLBP, non-painful and painful pressure stimulation as well as a light brushing stimulus were applied on the lower back, i.e., the problematic organ of those patients. Boendermaker et al. (2014) from our group have already applied such a pressure stimulation on the lower back in healthy subjects using functional magnetic resonance imaging (fMRI); they showed activity in sensorimotor areas like the S1 and SMA, besides others. Though they were only able to apply non-painful pressure, because painful pressure caused severe head motion artifacts in the fMRI data. To enable painful stimulation on the lower back but avoid motion artifacts due to head motion (Gervain et al., 2011; Boendermaker et al., 2014; Meier et al., 2014), functional near-infrared spectroscopy (fNIRS) was used in the current investigation to measure the cortical hemodynamic responses of oxy- and deoxyhemoglobin ([O_2_Hb] and [HHb]). fNIRS is a technique to determine changes in [O_2_Hb] and [HHb] in the human head non-invasively (Scholkmann et al., 2014b) and has the advantage of being more robust against motion artifacts than the fMRI. In addition, the application of fNIRS is more convenient, especially when measuring patients with CLBP, as the measurement can take place on a massage bench instead of the MR scanner bench, where lying in prone position is much more comfortable. The main disadvantage of fNIRS is its limited penetration depth, which limits the reachable regions. Nevertheless, important parts of the sensorimotor system are placed at the cortical surface, being therefore accessible by using fNIRS (Vrana et al., 2016). Based on these facts, the S1 and the SMA were assigned as regions of interest, both known to be active during non-painful pressure on the lower back (Boendermaker et al., 2014) and also involved in cortical sensorimotor pain-related processing in healthy subjects (Bushnell et al., 1999; Coghill et al., 1999; Xie et al., 2009; Yücel et al., 2015). The activity of the SMA is thought to reflect early motor planning/preparation in order to perhaps anticipate further possible damage, while S1 is thought to represent the sensory-discriminative aspect of pain processing (Bushnell et al., 1999; Xie et al., 2009).

To summarize, the main objective of the current study was to investigate cortical sensorimotor areas regarding potential alterations in sensorimotor processing, especially in the presence of pain. Therefore, patients with CLBP were investigated regarding their processing in two regions of interest (ROIs), the SMA and S1, after painful and non-painful pressure as well as light tactile stimulation on the lower back and subsequently compared with HC. We hypothesized to find altered cortical responses in [O_2_Hb] and/or [HHb] in at least one of the two areas compared to our healthy population, representing an affected sensorimotor processing due to the chronic pain impact in CLBP.

## Materials and Methods

### Subjects

14 patients with CLBP and 22 HC participated in this study. Two patients had to be excluded due to specific pain conditions (one with obvious degenerative alterations at the lumbar and thoracic level and one with a neuropathic pain component) and two healthy women due to a low signal-to-noise-ratio of the fNIRS signals (both had dark, thick and curly hair). Therefore, 12 CLBP patients (age: 32 ± 6.1 years; range: 24–44 years; nine women) and 20 HC (age: 33.6 ± 10.7 years; range: 22–61 years; eight women) were included in the final analyses.

The groups were age- and gender-matched (Pearson’s chi-squared-test for age and gender, *p* > 0.05). All subjects had no previous history of psychiatric or neurological disorders. Patients were included if they had non-specific LBP (neither traumatic nor inflammatory nor neuropathic origin) for longer than 6 months. The Pain Detect Questionnaire (Freynhagen et al., 2006) was utilized for pain assessment and to exclude neuropathic pain. Exclusion criteria for the HC were acute and/or recurrent back pain within the last 6 months and a past history of chronic pain episodes. Recruitment was done via online advertisement and word-of-mouth recommendation. Subjects were financially compensated for their participation and all provided written informed consent for the participation in the experiment. The study was approved by the Ethics Committee of the Canton of Zurich (KEK-ZH-Nr.2012-0029) and conducted in accordance with the Declaration of Helsinki.

### Functional Near-Infrared Spectroscopy Instrumentation

**Figure 1:**
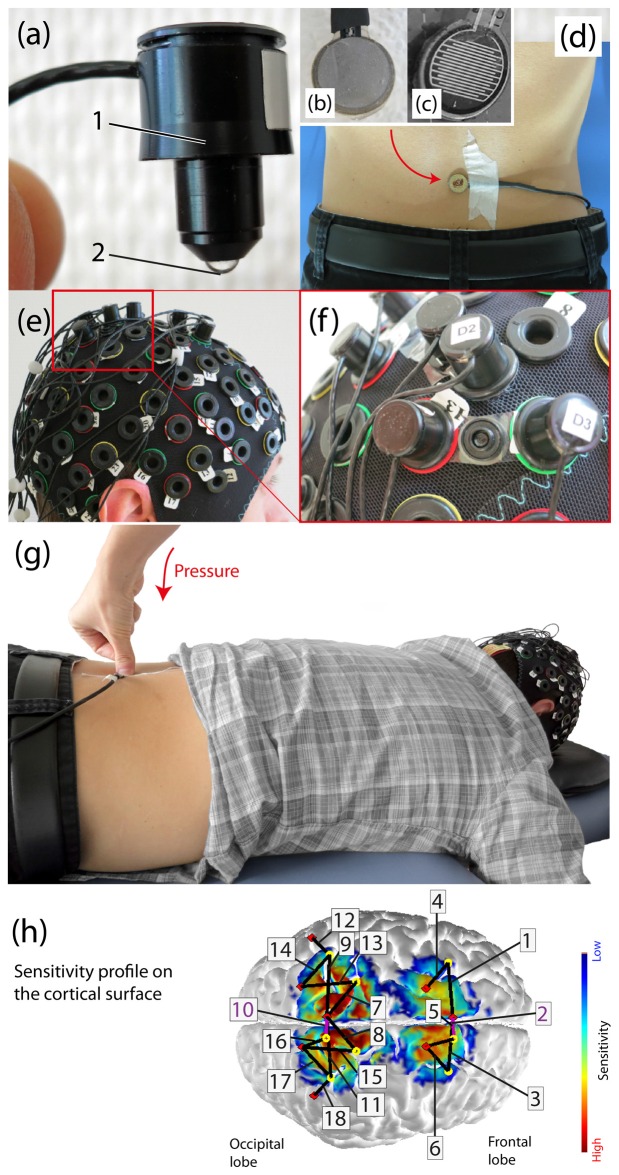
**Experimental setup. (A)** Example of an functional near-infrared spectroscopy (fNIRS) optode (1: enclosure of the optical fiber, 2: tip of the light emitting diode (LED)). **(B)** The force sensor from the bottom (this side is placed on the skin), **(C)** The inside of the force sensor. **(D)** Subject with the force sensor attached on third lumbar spinous process (L3), **(E)** Probe array on the subject’s head, **(F)** Fixation of a source and detector distance by using so-called distance-holders. **(G)** Visualization of the applied manual posterior-to-anterior (PA) pressure on the L3 in a subject lying in prone position on a massage bench, **(H)** Sensitivity profile [mm^−1^] of the probe array (calculated using AtlasViewer as implemented in HOMER2) including the detailed array of all channels. The sensitivity values are displayed in log10 units. The profile was calculated for 10 million photons.

For cortical measurement of hemodynamic and oxygenation changes a multi-channel continuous wave fNIRS imaging system (NIRSport, NIRx Medical Technologies LLC, Glen Head, NY, USA) operating at 760 nm and 850 nm was utilized. For data recording, the NIRStar Software 14.0 (NIRx Medical Technologies LLC, Glen Head, NY, USA) was employed. The probe setup was comprised of eight LED-sources (see Figure 1A) and eight detectors, forming an 18 multi-distant channel setup. The signal sampling rate was 7.81 Hz. Two ROIs were determined, i.e., the bilateral SMA and S1. The S1 area was set around the midline, in order to cover the back and trunk representation (Eickhoff et al., 2008). The SMA area was identified according to the craniocerebral topography within the international 10-20 system, as it was done previously (Steinmetz et al., 1989; Wang et al., 2007). For the elimination influences from the superficial tissue layers of the head (e.g., scalp blood flow changes; Saager et al., 2011), the channels 2 and 10 were set as short-separation channels with a source-detector separation of ~11 mm. The other channels had a source-detector separation ranging from 25 mm to 45 mm. These multiple source-detector distances served for monitoring different depths of tissues of the head. Textile EEG caps (EASYCAP, Herrsching, Germany) in three different sizes (i.e., having a circumference of 54 cm, 56 cm and 58 cm) were used in order to fix the LED-sources and detectors on the subject’s head. The probe arrangement was fixed in each of the caps in order to assure comparable probe placement over all subjects (see Figures 1E,F). Additionally, in order to assure appropriate placement of the probes for reaching the determined ROIs a spatial sensitivity profile of our specific probe placement was calculated based on the Monte Carlo photon migration forward modeling (Wang et al., 1995) using the AtlasViewer software (HOMER2 software package^1^; see Aasted et al., 2015; Figure 1H). This Monte Carlo photon migration modeling was calculated for 10 million photons, revealing that our probe setup is capable of measuring in the cerebral compartment of the S1 and SMA.

### Heart Rate Measurement

Heart Rate (HR) assessment was added as an additional physiological measurement. A Garmin Edge 500 device (Garmin Ltd., Schaffhausen, Switzerland; interval of measurement value update: 1 s) was employed and the HR belt was positioned at the lower sternum for the duration of the experiment.

### Experimental Design

Prior to the experiment, the patients with CLBP completed two questionnaires; the German version of the Roland-Morris Disability questionnaire (Wiesinger et al., 1999), which grades physical disability in everyday life activities, and the Pain Detect Inventory (Freynhagen et al., 2006) in order to assess pain quantity and quality.

The third lumbar vertebra (L3) was manually palpated while subjects stood erect with their back to the experienced examiner. Then subjects were seated on a chair for cap choice and adjustment. Head circumference was measured in order to determine the adequate cap size. For proper positioning, the Nasion-Inion length as well as the ear-to-ear distance were measured and proper positioning of the cap was adapted according to the international 10-20 positioning system (Chatrian et al., 1985). Optodes were fixed on the scalp (into the cap) after careful preparation, i.e., brushing away of hairs and applying a small amount of clear ultrasound gel (Aquasonic clear ultrasound gel, PARKER Laboratories, INC., Fairfield, NJ, USA) onto the scalp to ensure a good light coupling of the sensors/detectors (without strong light absorbers like hairs in between).

For the experiment, subjects were laid in the prone position on a massage bench. The entire experiment lasted 20 min starting with a baseline measurement of 5 min and continued afterwards with the application of three different stimuli in a pseudo-randomized order (no more than two equal consecutive trials). Each of the three stimuli was applied 15 times and each stimulus lasted 5 s with an inter-stimulus interval (ISI) of 15 s. HR was measured throughout the entire experiment with the Garmin device. In order to measure HR averages and maximum values, each trial was manually clocked. The pressure stimuli consisted of a painful and non-painful pressure exerted by the examiner’s thumb grip perpendicularly to the spinous process of the L3 (see Figure 1G), inducing a posterior-to-anterior (i.e., dorso-ventral) intervertebral movement (i.e., a PA-pressure stimulus). This manual technique is commonly used for assessment of spinal movement (joint-play) and spinal treatment (Snodgrass et al., 2006) in chiropractic therapy and physiotherapy. The painful stimulus was individually determined prior to the experiment by identifying the individual pressure-pain-threshold (PPT; Fischer, 1987; Ohrbach and Gale, 1989a,b). The pressure force was controlled by a force sensor (FlexiForce^®^Sensors, Teksan) which was attached at the spinous process of L3 (see Figures 1B–D). The sensor included an amplifier, transforming the resistive changes in an appropriate voltage signal, which was then digitalized by a micro-controller (1 kHz) and sent to a laptop where it was visible for the examiner throughout the experiment. In both groups, the PPT was assessed by slowly increasing the pressure force on the L3 vertebra until the PPT was reached (stimulus became clearly painful). This procedure was repeated 3–4 times and the values were then averaged for the final PPT. For individual PPTs see Table 1. The other pressure stimulus was a non-painful pressure force. In the HC group this non-painful pressure was standardized to 30 N. In the patients group, in most of the cases, the non-painful pressure force had to be lowered in order to assure non-painful stimulation. The determination of the non-painful pressure force, if lower than 30 N, was assessed with the same procedure as the PPT (3–4 time and then averaged). The third stimulus was a tactile stimulation by means of brushing strokes by the examinant’s thumb over the left *musculus erector spinae*.

**Table 1:** **Behavioral data for all 12 patients with chronic lower back pain (CLBP) who were participating in this study**.

	QUESTIONNAIRES: Patients with CLBP				
Condition	*Pain Detect current*	*Pain Detect ave 4 w*	*Pain Detect total*	*RMD*	*PPT*
Patient 01	3	4.3	3	2	75
Patient 02	2	4.3	5	4	40
Patient 03	0	3.3	2	4	60
Patient 04	3	5.3	8	11	65
Patient 05	1	2.7	3	1	57
Patient 06	3	4.3	5	5	55
Patient 07	2	4.3	5	2	25
Patient 08	3	3.7	1	3	45
Patient 09	0	3.7	14	6	20
Patient 10	2	4.0	11	1	20
Patient 11	2	4.3	7	1	40
Patient 12	2	3.3	6	4	32

MEAN	1.9	4.0	5.8	3.7	45
SD	1.1	0.7	3.8	2.8	18

*Detailed description of the values can be found below the table. Average values (MEAN) and standard deviations (SD) are indicated as well*. *Pain Detect*: Pain Detect Questionnaire, current pain PainDetect ave 4w: Pain Detect Questionnaire, average of current pain, average pain last 4 weeks and highest pain during the last 4 weeks (max. value: 10, represents highest pain level. *Pain Detect total*: Pain Detect Questionnaire, total score, maximal score 38; 0–12: probability for a neuropathic component <15%; 13–18: probability of neuropathic component is unclear; 19–38: probability for neuropathic component is >90%. *RMD*: Roland-Morris-Disability Questionnaire, 24 items, German version, maximal score 24 (the higher the score, the more disabled is the patient). *PPT*: Pressure Pain Threshold.

To summarize, the experimental protocol comprised three types of stimulation: (i) a painful PA-pressure according to the individual PPT (PAPain); (ii) a non-painful pressure with a force of ~30 N for HC and an individual non-painful pressure force for the patients (PAnP); and (iii) a tactile brushing stimulus (Brush).

In order to familiarize all subjects with the experimental conditions, all stimuli were performed once before the start of the experiment. Subjects were advised to keep their eyes open throughout the entire experiment and to avoid larger movements (e.g., no feet-tapping). Additionally, all subjects received the same easy cognitive task to prevent them from falling asleep. They were asked to count the quantity of one of the stimuli (they could freely choose which one they wanted to count). After the experiment, subjects were asked to report their counting results. If their answers were incorrect by more than two points, they were excluded from the analysis. One of the patients did not achieve the counting task correctly and was excluded from the analysis (remark: this patient was excluded anyway after the measurement as awareness emerged that he had a neuropathic pain component).

### Data Analysis and Statistics

#### fNIRS Signal Processing

Raw optical density (OD) data were analyzed using the NIRSLAB analysis software (NIRx Medical Technologies LLC, Glen Head, NY, USA). There, the OD data was converted into relative concentration changes of [O_2_Hb] and [HHb] by applying the modified Beer-Lambert-Law (Cope and Delpy, 1988; Cope et al., 1988). In order to correct for age-dependent changes of the skull anatomy and for varying source-detector separations, the differential path-length factor was calculated for each of the subjects, healthy and patients (Scholkmann and Wolf, 2013), including also the varying source-detector separations. Subsequently, datasets were transferred to Matlab (Mathworks, Natick, MA, USA version 2013b) and were filtered, i.e., the high-frequency noise of the signal was removed by applying a third degree Savitzky-Golay (SG; Savitzky and Golay, 1964) with a window length of 4 s (Schafer, 2011). The low-frequency trend was removed by applying the SG filter with a window length of 80 s and by subsequent subtraction of this low-frequency trend (Schafer, 2011). Both window length’s, i.e., 4 s and 80 s, were determined empirically. The main priority was the filtering of the low frequency Mayer waves (0.1 Hz) which are known to be able to impede the evidence of the task-related hemodynamic response. By using the SG filter instead of simple moving average filter or a FIR/IIR filter, the important information related to the evoked hemodynamic response was preserved, while non-related information was removed (Savitzky and Golay, 1964; Schafer, 2011). Subsequently, the datasets were segmented into single trials with a length of 5 s ± 3.9 s which corresponds to the stimulus duration (5 s) plus 3.9 s of pre-ISI and 3.9 s post-ISI. This procedure yielded 15 single trials per condition and subject. Each trial was then linearly de-trended in order to remove the slow physiological drift during each trial by applying a linear regression. Further, each trial was normalized by subtracting the median value of the 3.9 s long pre-ISI from the signal in each trial for removal of the intra-individual variance of the starting values. In order to remove physiological noise from the superficial layers of the head (i.e., the scalp blood flow), short-separation-regression (SSR; Saager and Berger, 2005) was applied. The channels 2 and 10 were used for the SSR with a source-detector distance of ~11 mm. SSR using the channel 2 was applied for the channels 1 and 3–6, while SSR using the channel 10 was applied for channels 7–9 and 11–18.

Subsequently, a median value was identified (from the middle 2.5 s of the 5 s lasting stimuli) from these 15 single trials, per condition, resulting in a block average (one value) per subject, channel and condition (PAPain, PAnP, Brush). Further, for the group level analysis, the grand-averages per condition and group were calculated by calculating the median of all the subjects within a group (20 in the HC group; 12 in the CLBP group). The variance was determined by calculating the standard error of the median (SEMed).

#### Statistical Analysis

Non-parametric statistical tests were used throughout the analysis due to not normally distributed data. At single-subject level, Wilcoxon signed-rank tests were applied per channel and condition in order to check for significance of the [O_2_Hb] and [HHb] responses. At group-level, two different analysis approaches were used. A first classic approach, where all channels in all conditions and subjects were included (*Analysis_All*). In this analysis the results at single-subject level were not considered. In contrast, the second approach included only those channels in the group-level analysis, which were already showing statistical significance at single-subject level (*Analysis_Responders*). Therefore, only channels which showed a neural or systemic task-related change were included, while channels which did not show any response were excluded from the group analyses in order to enhance specificity and decrease the false-positive rate (Tachtsidis and Scholkmann, 2016; Vrana et al., 2016). Both approaches are provided for all statistical within-group analyses.

At group-level, first within-group (for the healthy and patient group separately) tests were conducted. The main effect of condition was calculated by using a Friedman-test. In these tests, the results were corrected for multiple comparisons by the false discovery rate (FDR) correction, following the Benjamini and Hochberg (1995) procedure implemented in Matlab (*q* < 0.05). Subsequently, *post hoc* paired Wilcoxon rank-sum tests were calculated in order to assess significant differences between these three different conditions (FDR-corrected, *q* < 0.05). The grand-averages (averaged medians) were tested for significance by applying Wilcoxon signed-rank tests per channel and corrected for multiple comparisons (FDR, *q* < 0.05). In addition, a Spearman correlation analysis was calculated comparing the individual PPTs of all subjects (patients with CLBP and HC) with the magnitude of their hemodynamic responses ([O_2_Hb]) in each channel in order to disentangle a possible relationship (FDR-corrected, *q* < 0.05).

Between-group comparisons (HC vs. CLBP patients) were calculated by Wilcoxon signed-rank tests per condition and channel (FDR-corrected, *q* < 0.05). Additionally, we conducted an analysis which compared the block averages (median from all 15 single trials per condition, channel, and subject) from every single patient to the grand-averages of the HC (median from all 20 HC per condition and channel), in order to analyze how single patients’ data deviated from the data of a “normal” healthy population. Wilcoxon signed-rank tests were used for this purpose. The median values per condition (PAPain, PAnP, Brush), channel and patient were compared to the grand-averages of all 20 HC, per channel (FDR-corrected, *q* < 0.05). This procedure was repeated for all conditions in all patients, and across both [O_2_Hb] and [HHb].

#### Heart Rate Analysis

HR data was exported from the Garmin Edge500 device to Excel (Microsoft, Redmond, WA, USA). Clocked rounds yielded averaged *HR_mean_* values (per each stimulus, 5 s; and per each ISI, 15 s) and *HR_max_* values per stimulus and ISI. The ISIs were divided into pre-ISI (the ISI before a specific stimulus) and the post-ISI (the ISI after a specific stimulus). For each condition, the mean, the standard deviation (SD) and the standard error of the mean (SEM) were calculated for pre- and post-ISI for *HR_max_* and *HR_mean_*. In order to answer the question whether there was a significant difference between HR during the stimulus and during the subsequent post-ISI, three paired Wilcoxon sign-rank test were calculated (e.g., HR during PAPain vs. HR during post-ISI of PAPain; *q* < 0.05, FDR-corrected). To compare the HRs of the three different conditions, the difference between the *HR_max_* during the stimulus and the *HR_max_* during the post-ISI was calculated yielding Δ*HR_max_*. This Δ*HR_max_* (per condition) was calculated using a paired Friedman-test (*q* < 0.05, FDR-corrected).

## Results

### Behavioral Data

All behavioral data, including individual PPTs of the patients are listed in the Table 1. Patients with CLBP were revealed to have significantly lower PPTs compared to the HC (mean: 57 N, SD: 7.3 N; *p* < 0.05).

### Stimulus-Evoked Hemodynamic Responses

On the single subject level, in both [O_2_Hb] and [HHb], similar percentages of significant channels were identified within the 12 patients with CLBP. For the painful condition, 48% of channels showed significant hemodynamic changes ([O_2_Hb] and [HHb]). For the non-painful pressure condition percentage of active channels was slightly higher for [O_2_Hb] (53%) than for [HHb] (46%). The brushing condition only yielded between 4–5% for both, [O_2_Hb] and [HHb]. For the HC, percentages were higher with larger differences between [O_2_Hb] and [HHb]. For [O_2_Hb] percentages of significant channels were 73% (painful condition), 62% (non-painful condition) and 16% (brushing condition). For [HHb], percentages of significant channels were 50% (painful condition), 55% (non-painful condition) and as well 16% (brushing condition).

**Figure 2:**
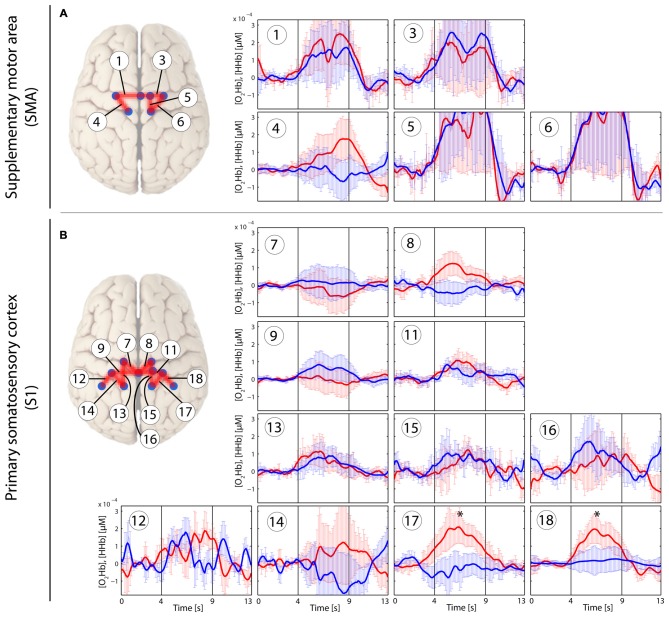
**Stimulus-evoked hemodynamic responses in patients with chronic lower back pain (CLBP) elicited by a painful PA-pressure stimulus (PAPain) according to the individual pressure-pain-threshold (PPT; PAPain).** Changes in [O_2_Hb] (red) and [HHb] (blue) are depicted as changes in the median concentration. The two vertical lines within the graph represent the stimulus on- and offset (duration: 5 s). Error bars represent the standard error of the median. **(A)** Supplementary motor area (SMA), **(B)** primary somatosensory cortex (S1). An asterisk represents a statistically significant hemodynamic response (*q* < 0.05, corrected for multiple comparisons using false discovery rate [FDR]).

**Figure 3:**
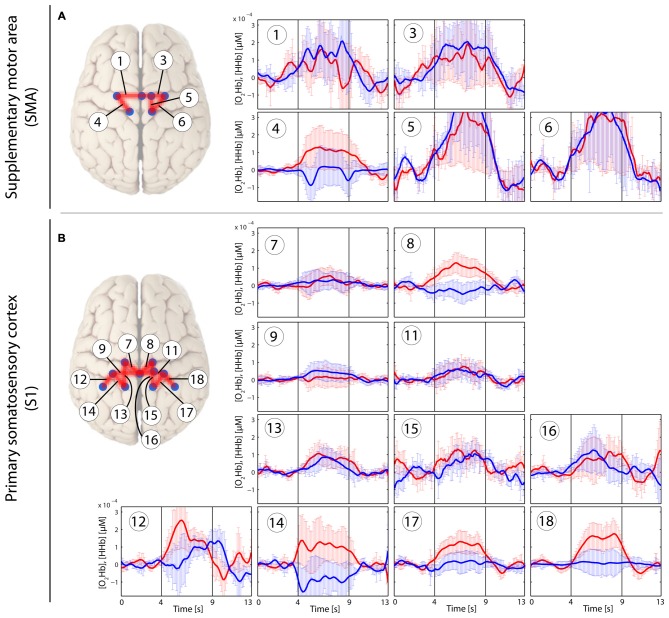
**Stimulus-evoked hemodynamic responses in patients with CLBP elicited by an individual non-painful stimulus with a pressure force for each patient (PAnP).** Changes in [O_2_Hb] (red) and [HHb] (blue) are depicted as changes in the median concentration. The two vertical lines within the graph represent the stimulus on- and offset (duration: 5 s). Error bars represent the standard error of the median. **(A)** Supplementary motor area (SMA), **(B)** primary somatosensory cortex (S1). An asterisk represents a statistically significant hemodynamic response (*q* < 0.05, corrected for multiple comparisons using false discovery rate [FDR]).

**Figure 4:**
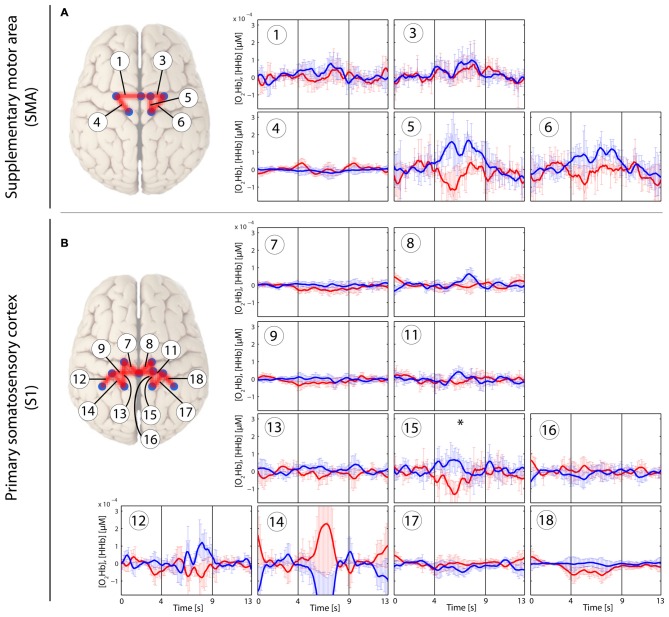
**Stimulus-evoked hemodynamic responses in patients with CLBP elicited by a tactile brushing stimulus (Brush).** Changes in [O_2_Hb] (red) and [HHb] (blue) are depicted as changes in the median concentration. The two vertical lines within the graph represent the stimulus on- and offset (duration: 5 s). Error bars represent the standard error of the median. **(A)** Supplementary motor area (SMA), **(B)** primary somatosensory cortex (S1). An asterisk represents a statistically significant hemodynamic response (*q* < 0.05, corrected for multiple comparisons using false discovery rate [FDR]).

In the patients with CLBP, the main effect of condition (“*Analysis_All”*) in [O_2_Hb] at group level was yielded only by three channels in the right S1 compared to a bilaterally represented main effect in the HC group. Additionally, the HC revealed a main effect within the SMA (right; *q* < 0.05, FDR-corrected), but not for patients with CLBP (for detailed results see Tables 2A,B). Grand-averages per condition and channel are shown in Table 3. Additionally, grand-averages for the patients with CLBP are also displayed in Figures 2–4 (calculated using the *Analysis_All* approach), visualizing the dynamics of the hemodynamic responses and their significance against baseline. The dynamics of the hemodynamic responses (against baseline) of the HC group have been already published elsewhere (Vrana et al., 2016). Patients with CLBP exhibited significant changes in [O_2_Hb] only in the painful condition in right S1 and also in one channel in the brushing condition, while HC exhibited significant changes in [O_2_Hb] in both pressure conditions as well as both ROIs (for detailed results see Table 4). Further, *post hoc* paired Wilcoxon rank-sum tests were conducted, revealing that in patients with CLBP, only the painful vs. brushing condition yielded significant difference within the right S1 for [O_2_Hb]. In contrast, HC revealed significant differences in both, painful and non-painful pressure condition and also in both ROIs (for [O_2_Hb] (Table 3). [HHb] yielded no significant (FDR-corrected) results for either patients with CLBP or the HC. Further, the Spearman correlation analysis comparing individual PPTs with the hemodynamic responses for all subjects and channels yielded no correlation effect in any of the channels (*q* > 0.05).

**Table 2:** **Main effect of condition for patients with CLBP and healthy controls (HC; A) oxyhemoglobin ([O_2_Hb]) and (B) deoxyhemoglobin ([HHb])**.

		[O_2_Hb]_All_				[O_2_Hb]_Responders_			
		CLBP		HC		CLBP		HC	
Region	Channels	*q* (FDR)	χ^2^	*q* (FDR)	χ^2^	*q* (FDR)	χ^2^	*q* (FDR)	χ^2^
**A**
SMA	1
	3
	4							0.0304	8.042
	5			0.016	13.3			0.016	11.645
	6			0.02	11.1			0.03	8.444
S1	7							0.0627	6.136
	8
	9			0.064	7.5			0.0304	7.875
	11			0.086	6.3			0.03	9.22
	12			0.048	8.4			0.000	15.460
	13
	14			0.08	6.7			0.03	8.667
	15	0.032	10.167					0.048	6.821
	16							0.03	8.522
	17	0.032	10.5	0.02	10.8			0.000	15.216
	18	0.032	12.167	0.016	12.1			0.03	9.418

**B**		**[HHb]_All_**				**[HHb]_Responders_**			

SMA	1			0.064	11.1				
	3
	4
	5							0.096	10.383
	6
S1	7
	8
	9
	11
	12
	13
	14
	15
	16
	17
	18

*Channels 1–6 belong to the bilateral supplementary motor area (SMA) while channels 7–18 belong to the bilateral primary somatosensory cortex (S1). The subscript “All” means that all, responding and non-responding channels, were included for analysis whereas in the “Responders” group only the responding channels were included. Shown are significant channels as well as tendencies, both corrected for multiple comparisons by applying false discovery rate (FDR) correction (q < 0.05)*.

**Table 3:** ***Post hoc* comparisons for patients with CLBP and HC of relative oxyhemoglobin ([O_2_Hb]) changes between painful pressure (=PAPain), non-painful pressure (=PAnP) and brushing (=Brush)**.

		[O_2_Hb]_All_				[O_2_Hb]_Responders_			
		CLBP		HC		CLBP		HC	
Region	Channels	*q* (FDR)	*Z*	*q* (FDR)	*Z*	*q* (FDR)	*Z*	*q* (FDR)	*Z*
**(A)** PAPain vs. PAnP	1
	3
	4
	5
	6
	7
	8
	9
	11
	12
	13
	14
	15
	16
	17
	18

**(B)** PAPain vs. Brush	1
	3
	4							0.064	−2.197
	5			0.0107	−3.024			0.0213	−3.051
	6			0.0107	−3.136			0.0512	−2.411
	7							0.0768	−1.977
	8
	9			0.0613	−2.277			0.068	−2.118
	11	0.06	−2.432	0.024	−2.725			0.0512	−2.497
	12			0.064	−2.203			0.0213	−2.844
	13
	14			0.080	−2.053			0.0768	−2.017
	15	0.032	−2.746						
	16			0.0316	−2.315			0.0613	−2.275
	17	0.032	−2.981	0.0107	−3.061			0.0213	−2.934
	18	0.032	−2.903

**(C)** PAnP vs. Brush	1
	3
	4							0.0526	−2.395
	5			0.04	−2.688			0.0526	−2.900
	6	0.0736	−2.275	0.04	−2.427			0.0526	−2.312
	7							0.062	−2.158
	8	0.0747	−2.197						
	9			0.04	−2.613				
	11		−1.883	0.04	−2.464			0.0526	−2.366
	12	0.0640	−2.589	0.04	−2.501			0.0526	−2.551
	13		−1.883						
	14
	15	0.064	−2.746						
	16			0.0846	−2.091				
	17	0.0736	−2.275	0.016	−3.360			0.0526	−2.551
	18	0.0736	−2.275					0.0526	−2.271

***(A)** PAPain vs. PAnP, **(B)** PAPain vs. Brush and **(C)** PAnP vs. Brush. The subscript, “All” means that all, responding and non-responding channels, were included for analysis whereas in the “Responders” group only the responding channels were included. Results are false discovery rate (FDR) corrected (q < 0.05)*.

**Table 4:** **Comparisons for patients with CLBP and HC of all three conditions against baseline for oxyhemoglobin ([O_2_Hb])**.

		[O_2_Hb]_All_				[O_2_Hb]_Responders_			
		CLBP		HC		CLBP		HC	
Region	Channels	*p*	*q* (FDR)	*p*	*q* (FDR)	*p*	*q* (FDR)	*p*	*q* (FDR)
**(A)** PAPain vs. Baseline	1
	3
	4							0.0161	0.0368
	5			0.0004	0.0064			0.0061	0.0098
	6			0.001	0.008			0.0081	0.0314
	7
	8
	9			0.0333				0.0342	
	11	0.0324		0.0152	0.0347			0.0098	0.0314
	12			0.01	0.0347			0.0034	0.0208
	13
	14			0.0111	0.0347			0.0259	
	15	0.0640
	16			0.0137	0.0347			0.0122	0.0325
	17	0.0024	0.0192	0.004	0.0213			0.0039	0.0208
	18	0.0024	0.0192					

**(B)** PAnP vs. Baseline	1
	3
	4			0.0152	0.0347			0.0098	0.0499
	5			0.0009	0.0142			0.001	0.0156
	6	0.0269		0.0072	0.0288			0.0137	0.0499
	7							0.0353	
	8
	9			0.0124	0.0347			
	11			0.0479				0.0156	0.0499
	12			0.0152	0.0152			0.0134	0.0499
	13
	14							0.0494	
	15							
	16			0.0045	0.0288			
	17	0.0068		0.0057	0.0288			0.0269	
	18	0.0269				0.0156			

**(C)** Brush vs. Baseline	1
	3
	4
	5
	6
	7
	8
	9	0.0342							
	11
	12
	13
	14
	15	0.0024	0.0384						
	16	0.0342							
	17
	18

*Deoxyhemoglobin ([HHb]) did not reveal any significant difference in both analyses (“All” and “Responders”). PAPain, painful pressure; PAnP, non-painful pressure and Brush, brushing. The subscript “All” means that all, responding and non-responding channels, were included for analysis whereas in the “Responders” group only the responding channels were included. Results are false discovery rate (FDR) corrected (q < 0.05)*.

The *Analysis*_*Responders* approach did not show any significant results in the CLBP group for any of the analyses. In the HC group, the *Analysis_Responders* approach showed similar results to the *Analysis_All* approach (for detailed results see Tables 2–4).

**Figure 5:**
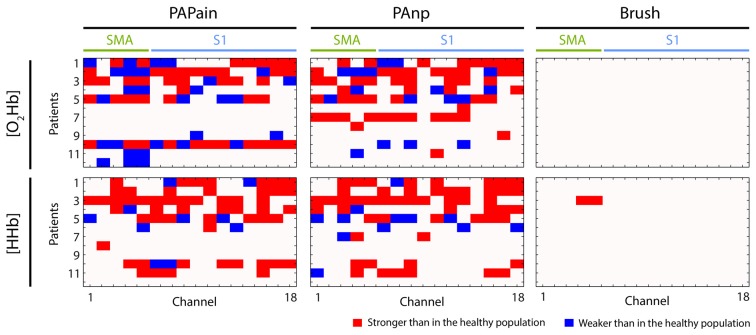
**Visualization of individual hemodynamic responses (depending on the channel, type of stimulus, oxy- or deoxyhemoglobin ([O_2_Hb] or [HHb])) in patients with CLBP (*n* = 12) that were statistically significantly different to the group average of hemodynamic responses obtained from the healthy population.** The direction of deviation is color-coded (red: stronger, blue: weaker). PAPain, painful pressure; PAnp, non-painful pressure; Brush, brushing; SMA, supplementary motor area; S1, primary somatosensory cortex.

Finally, a direct comparison between groups did not yield any significant results for any of the three conditions or for [O_2_Hb] or [HHb] (only *Analysis_All* approach). Only uncorrected results reached in some channels a significant difference (*p* < 0.05). The comparison of single patients with the normal population (grand-averages) revealed high inter-subject variability across the patients group (*q* < 0.05, FDR-corrected; see Figure 5).

### Heart Rate Changes

*HR*_max_ and *HR_max_-post* elicited a similar pattern in both groups and across all three conditions (see Table 5). In both groups, a clear effect of condition was observed, as post-ISI HR_max_ (*HR_max_-post*) were in all conditions significantly higher than *HR_max_* during the all conditions. However, no statistically significant difference in *HR_max_* or *HR_max_-post* was observed between the three conditions (for all results *q* < 0.05, FDR-corrected). HR_mean_ values did not show any significant differences, neither between the conditions nor between post-ISI conditions (*q* < 0.05, FDR-corrected).

**Table 5:** **Maximal heart rate (*HR*_max_) values and *HR*_max_ post-stimulus values (*HR*_max_-*post*) in HC and patients with CLBP per condition (PAPain, painful pressure; PAnP, non-painful pressure; Brush, brushing)**.

	Patients with CLBP		HC	
Condition	*HR_max_*	*HR_max_-post*	*HR_max_*	*HR_max_-post*
PAPain	63.3 ± 10.9	64.1 ± 11.0	62.0 ± 7.6	62.7 ± 7.7
PAnP	63.2 ± 11.0	63.9 ± 11.0	62.1 ± 7.3	62.8 ± 7.4
Brush	63.3 ± 10.8	64.1 ± 11.3	62.3 ± 7.3	62.8 ± 7.3

*Values are presented with ± SD (standard deviation)*.

## Discussion

The main results of this study can be summarized as follows: (i) direct comparison between groups yielded no significant differences regarding the hemodynamic response, neither in the SMA nor in the S1 for any of the conditions; (ii) within-group results, however, point to the SMA as ROI for future investigations regarding maladaptive sensorimotor processing in patients with CLBP; and (iii) in addition, we found a high inter-subject variability within the CLBP cohort which could point to a subgrouping within the patients group.

### Behavioral Measures

The average pain rating of the last 4 weeks yielded a moderate level of pain across the patients group (Table 1). Referring to the Pain Detect questionnaire (Freynhagen et al., 2006), the scores revealed to be low, representing a minor probability of a neuropathic pain component, which corroborated that the patients cohort suffered from non-specific CLBP. Both disability questionnaires yielded low disability scores, despite moderate pain levels. This could point to a high self-efficacy of the patients according to the proposed disability model of Arnstein et al. (1999), which proposes self-efficacy as a mediator for disability.

The PPTs in the patients with CLBP were lowered compared to the HC. This finding is in accordance with several other reports showing specific and unspecific (regarding the site of stimulation) hyperalgesia in patients with CLBP (Giesecke et al., 2004; Giesbrecht and Battié, 2005; O’Neill et al., 2007; Kobayashi et al., 2009; Wand et al., 2011). In particular, this manual pressure represents a very familiar sentiment of pain for CLBP patients, eliciting a rather deep and dull pain sensation. Pressure pain activates both Aδ- and C-fibers simultaneously, beside all the mechanoreceptors, while thermal pain remains rather superficial, elicits a sharp pain sensation and is mediating first Aδ-fibers and subsequently the C-fibers (Olesen et al., 2012; Reddy et al., 2012). In addition, manual pressure on the spinous process represents an established clinical tool for physicians and therapists in order to assess the actual condition of a patient (Snodgrass et al., 2006). Therefore, it might be of interest what such a stimulation provokes within the cortical areas.

### Between-Group Results: Patients With CLBP vs. Healthy Controls

The main comparison did not yield any significant difference between the patients with CLBP and HC. Therefore, it seems that painful pressure at the lower back is not differentially processed within a substantial part of the sensorimotor network, the SMA and S1. However, a closer look at the data is needed for an appropriate interpretation.

Contrary to our hypothesis, there was no statistically relevant difference between the relative changes in [O_2_Hb] and [HHb] after painful and non-painful pressure stimuli applied at the lower back in patients with CLBP compared to the HC. However, this non-significant result might also be driven by the high inter-subject variability of hemodynamic responses that have been observed in patients with CLBP. The grand-averages yielded a highly enhanced variability by means of standard error in almost all 16 channels in both, [O_2_Hb] and [HHb]. As the correlation analysis has shown that this inter-subject variability was not intensity-specific due to lower thresholds, but rather indicated a subgrouping within the patients group. The comparison of single patients against the healthy population confirmed the large inter-subject variability within the patients group. Some patients exhibited significantly higher responses in one or both ROIs, whereas others showed significantly decreased activity within one or both ROIs (Figure 5). This finding point to an inhomogeneous patient group, despite several similar characteristics (see Table 1). Indeed, reports about subgrouping in patients with CLBP do exist, at least at behavioral level. Keefe et al. (1990) investigated large populations of patients with CLBP and found four subgroups due to several behavioral factors like the overall magnitude of pain behavior, levels of guarding, bracing and rubbing. Another investigation yielded evidence about sub-classifications of patients with CLBP due to different patterns of superficial trunk muscle activation (Dankaerts et al., 2006). Although the large distribution of PPTs points to a potential behavioral and/or peripheral subgrouping, the utilized behavioral measures were not sufficient to further investigate such a potential sub-grouping and hence further research is needed to elucidate whether already known subgrouping factors like pain-related behavior (Keefe et al., 1990) or muscle activity at the lower back (Dankaerts et al., 2006) in patients with CLBP may also contribute to an effect on cortical sensorimotor processing.

The HR-analysis revealed similar results in both groups, showing an effect of the condition compared to the ISI’s, however no difference between the HR during the three different stimuli was observed. First, this result underlines the importance of the short separation regression analysis for the control of superficial hemodynamic changes which might be associated with a task-related increase of HR (Boudreau et al., 2010a,b; Gregg et al., 2010; Saager et al., 2011). Second, the missing difference between the three stimulations might indicate that the painful stimulation was too “low” (only PPT) and/or too short to induce significant changes regarding HR compared to the other stimulations.

### Within-Group Results of Patients With CLBP and HC

**Figure 6:**
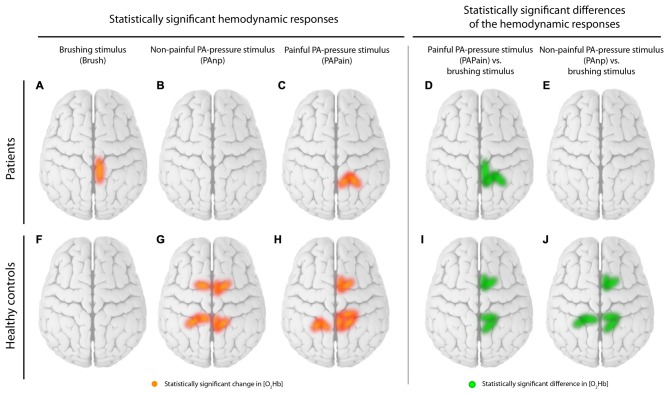
**Visualization of the within-group results.** Statistically significant channels for [O_2_Hb] are displayed separately for patients and healthy controls (HC). **(A)** Brushing stimulus vs. baseline, **(B)** Non-painful posterior-to-anterior (PA) stimulus (PAnP) vs. baseline, **(C)** PAPain vs. baseline, **(D)** PAPain vs. the brushing stimulus and finally **(E)** PAnP vs. the brushing stimulus. Therefore, red lines display statistically significant channels in comparisons vs. baseline per group, while green lines display significant channels in comparisons of the conditions against each other per group (*q* < 0.05, corrected for multiple comparisons using false discovery rate [FDR]).

**Figure 7:**
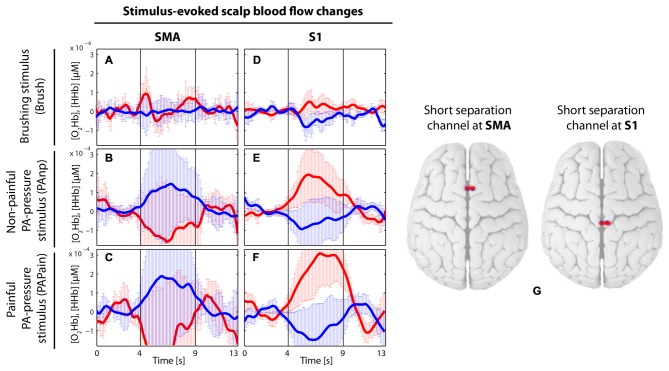
**Visualization of the grand average of evoked hemodynamic changes in the two short-distance channels (channel 2 and 10), which were used in the main analysis to remove the signals from the extracerebral layers (of no interest) by short separation regression. (A–C)** Short-distance channel in the supplementary motor area (SMA) in the brushing condition **(A)** in the non-painful condition **(B)** and in the painful condition **(C)**. **(D–F)** Short-distance channel in the primary somatosensory cortex (S1) in the brushing condition **(D)** in the non-painful condition **(E)** and in the painful condition **(F)**. Red thick lines display the [O_2_Hb] median concentration change; blue thick lines display the [HHb] median concentration change; the two vertical lines within the graph represent the stimulus on- and offset (duration: 5 s). Error bars represent the standard error of the median. **(G)** Visualization of the placement of the two short-distance channels.

First, comparing the within-group results of both groups and for both pressure stimulations (PAPain and PAnP) a main difference was found regarding the [O_2_Hb] relative changes within the SMA (for the values see Table 4). While in the HC the [O_2_Hb] in the SMA was significantly activated at least unilaterally (right), the patients with CLBP did not show any (FDR-corrected) relative changes in this area in any of the three different stimulations (for visualization see Figure 6). Interestingly, this is not the first time that patients with CLBP fail to show a hemodynamic response in the SMA. An fMRI study investigating visually guided motor imagery, conducted by our group, has also shown decreased or absent SMA activity during the motor imagery tasks in patients with CLBP compared to HC (Vrana et al., 2015). Furthermore, there is evidence for an involvement of the SMA in the processing of painful stimuli as it has been shown to be regularly active in pain-related processing (Apkarian et al., 2005) and its activity seems to correlate with pain intensity (Coghill et al., 1999). Nevertheless, because the activity of the SMA did not reach significance in the direct comparison between groups, this within-group result has to be interpreted very carefully and might serve only as a tendency and urge to consider this region for future investigations. However, a very interesting aspect is the activation pattern of the SMA-channels (channel 1–6) in particular. The patient group shows large hemodynamic responses with both [O_2_Hb] and [HHb] increasing, while the HC group revealed the expected response with [O_2_Hb] increasing and [HHb] staying stable or decreasing. With the present datasets, we are not able to disentangle this phenomenon, however we hypothesize that the patients possibly developed a higher systemic reaction/arousal to the pressure stimulations on the lower back compared to the HC group. The parallel increase in [O_2_Hb] and [HHb] refers to an increase in the total hemoglobin [tHb], i.e., the blood flow/volume. Since such a change is not evident in the scalp blood flow (see Figure 7) the observed hemodynamic responses in the SMA-channels are much likely the result of an interplay between neurovascular-coupling and a change in systemic blood flow/volume in the cerebral compartment. Such a change is mainly mediated by changes in blood pressure and/or respiration. This reasoning is supported by the fact that the other channels (i.e., the S1-related channels) also display this characteristic increase in [tHb], indicating that the stimulus-evoked increase in blood flow/volume is happening in the cerebral compartment of the whole head (an effect known to be caused by changes in systemic physiology, i.e., blood pressure and/or respiration).

Second, the S1 revealed robust bilateral [O_2_Hb] changes in the HC group, again in both pressure stimulations, while the CLBP group exhibited significant hemodynamic changes in only the right S1 and only after painful pressure (for visualization see Figure 6). As the spine has been previously shown to be represented bilaterally in the S1 (Penfield and Boldrey, 1937; Rasmussen and Penfield, 1947; Eickhoff et al., 2008), we would have expected bilateral activation. Additionally, a previous investigation using the same stimulus modality but only non-painful pressure has indeed reported bilateral activity in the S1 healthy subjects (Boendermaker et al., 2014). Moreover, in patients with CLBP, and also in more pain-sensitive healthy subjects, investigations are pointing to a rather enhanced cortical activity in the S1 during painful stimulation than reduced activity (Coghill et al., 2003; Giesecke et al., 2004). However, what is striking in the current data is the high data variability in the CLBP group, as best seen in Figure 5. This might explain the unilateral results in the S1, so that bilateral activity was simply lacking significance due to high SDs.

A possible habituation and/or sensitization effect in the hemodynamic responses in the healthy population in our study was analyzed in our previous article (Vrana et al., 2016). Neither a habituation nor a sensitization effect at group level in this healthy population was found for our pressure stimulation on the lower back. In comparison to the results of Yücel et al. (2015) who showed a clear habituation effect in the painful condition, our interpretation of our results was that the intensity difference between the noxious stimuli of Yücel et al. (2015) and our painful stimuli (which were significantly lower) might explain the missing habituation effect in our experiment. Besides, the kind of the stimulus (electrical vs. mechanical) and different duration of the ISI (Sarlani and Greenspan, 2002) may also have played a major role in the temporal summation of our mechanically induced pain stimulus. In addition, a previous study has indicated that patients with CLBP might show a reduced or absent habituation effect to painful stimulation in contrast to the healthy population or other pain patients (Flor et al., 2004). Thus, for the present study a habituation analysis was not performed.

Additionally, there are a few analytical aspects which are worth mentioning. First, the results from the two short-distance channels, which were incorporated into the analysis in order to regress the extracerebral signal of no interest (short-separation regression, for detailed description see the “Materials and Methods” Section), are corroborating how important this correction is for fNIRS measurements. In Figure 7, grand averages of both short-distance channels, one located in the SMA and the other in the S1, are visualized before the short-separation regression. The short-distance channel in the S1 is showing a clear [O_2_Hb] increase with a simultaneous decrease of [HHb] for both pressure stimulations, indicating a task-related response within the extracerebral layers, while the short-distance channel in the SMA does show the opposite, a [O_2_Hb] decrease and [HHb] increase for both pressure stimulations. These results could be interpreted as follows: Extracerebral layers are showing an intensified task-related hemodynamic response, which however is not directly related to the intracerebral, i.e., cortical hemodynamic, response, which is the only one of interest for the current objectives. Therefore, it is of immense importance to regress the impact of the extracerebral layers out of the whole hemodynamic response in order to avoid a falsification of the final result and interpretation (Kirilina et al., 2012; Tachtsidis and Scholkmann, 2016).

Further, the “*Analysis_Responders”* did not reveal any significant changes in CLBP patients, contrary to the HCs. This might be explained by low power in the “*Analysis_Responders*” approach due to smaller numbers of responding channels (45–50% of channels were “responding” yielding approx. 5–6 channels [12 subjects]). Therefore, as such small numbers may decrease the statistical power, we recommend application of this “*Analysis_Responders”* approach only in the case of sufficient power and together with a classical analysis approach to enable comparisons.

Another analytical aspect is the absence of significant changes in [HHb] in both, HC and patients with CLBP. This might be explained by the lower signal-to-noise-ratio of [HHb] compared to [O_2_Hb] and its smaller effect size (Fox and Raichle, 1986). Interestingly, the analysis comparing single patients against the healthy population revealed larger [HHb] changes in the patients compared to the HC, however also with correspondingly higher SD. Such large changes might then point to a systemic reaction, i.e., a large increase in cerebral blood flow due to a stress reaction, rather than reflecting neural activity (Tachtsidis and Scholkmann, 2016).

### Limitations

One limitation of the study is that there is a lack of representative behavioral biomarkers in order to investigate the potential subgrouping within the patients group. For a further study, we therefore propose to include not only questionnaires but also clinically relevant measures regarding the muscular activity status and pain-related behavior.

Further, we would propose to measure the electrodermal activity (related to the activity of the autonomic nervous system; Bray and Moseley, 2011; Yücel et al., 2015) or end-tidal CO_2_ concentration (Giesbrecht and Battié, 2005; O’Neill et al., 2007; Scholkmann et al., 2013, 2014a; Holper et al., 2014) for more accurate assessment of systemic changes rather than the HR measurements used in this study.

### Conclusion

The current investigation revealed that direct comparison between patients with CLBP and HC yielded no group difference indicating that the two investigated sensorimotor areas do not show specificity for CLBP during pressure stimulation at the lower back at least at first sight. However, high inter-subject variability was found within the CLBP group, which might have masked potential differences, and is indicative of a potential sub-grouping across patients with CLBP. High inter-subject variability in the case of pathology emphasizes the importance of single-subject analyses in order to look at individual features of pathology, as it has been recently proposed (Luomajoki and Moseley, 2011). This may be important especially for future investigations in order to reveal and classify potential sub-grouping with regard to behavioral, peripheral and central changes in patients with CLBP.

## Author Contributions

AV and FS: conception and design, analysis and interpretation of the data, drafting the work, final approval of the version and agreement to be accountable for all aspects of the work. MLM, SH-B and BKH: conception and design, revising it critically for important intellectual content, final approval of the version and agreement to be accountable for all aspects of the work.

## Conflict of Interest Statement

The authors declare that the research was conducted in the absence of any commercial or financial relationships that could be construed as a potential conflict of interest.

## Footnotes

^1^http://www.nitrc.org/projects/homer2
